# Pure Food and Efficient Living*Written for Woman’s Department, *Texas Medical Journal*.

**Published:** 1913-12

**Authors:** J. S. Abbott

**Affiliations:** Food and Drug Commissioner


### Pure Food and Efficient Living.*

BY J. S. ABBOTT, FOOD AND DRUG COMMISSIONER.

The greatest business in this world is raising babies—if not our own, then some one else's. It is a business which will never suffer for lack of raw material. The supply is always equal to the de-

*Written for Woman's Department, *Texas Medical Journal.*

mand, and the finished product never quite comes up to our expectations. We therefore continuously and laboriously strive to produce a manhood and a womanhood of tomorrow superior to that of today—mentally, morally, religiously, and physically. Whether or not it is the part of wisdom for society to try to save the life of every child born into its midst is a question which I shall not attempt to answer. It is settled individually for me and for every other man who has children. It is the sacred duty of society, however, to protect me in every faithful effort of mine to make my baby a valuable addition to society. The better the animal I make out of my baby, the better chance it will have of becoming an efficient citizen. There is no such thing as a sound mind in an unsound body. There is nothing so ugly and useless as a diseased body. There is nothing so pitiful as a half starved or poorly nourished child. Life is the most interesting thing in the world—from a squalling colicky kid to a prima donna. Death is the saddest thing in the world. And still that which makes for life or death has until recently caused us little concern—I mean, food.

The consumer wants a snowy white flour, not inquiring what it takes to produce the desired color, and the miller does the most natural thing in the world to do—he bleaches it with the oxides of nitrogen made by the "make" and "break" of an electric current or by passing an electric current through nitric acid. The consumer wants her pickles crisp and brittle, and the manufacturer adds alum to them. The consumer wants her English peas to be green, very green, and the little Frenchman paints them with copper sulphate or indigo-disulpho acid. She does not know that paint is injurious to the inside skin as well as to the outside skin. The consumer wants her dried apples to have a whitish faded color in place of a golden brown like they used to be back in the good old days when you men and I were young, and the manufacturer bleaches them with the oxides of sulphur which the butcher uses to take the stink out of decayed meat scraps used in making sausages. The same thing is used to bleach molasses and syrup to a pale straw color. The consumer wants a cherry to look red and the manufacturer paints it with Bismarck brown, erythrosin or something else. The consumer wants "blue points," and she would accept as such a Texas oyster six inches long provided it were blue around its gills and palps from the micro-organism which our oysters sometimes feed upon. We call for Rockyford cantaloupes although it is a fact that the Texas cantaloupe is a superior melon. We call for sugar corn, although we do not know it by its flavor, consistency, or grain morphology, and the manufacturer adds a little sugar or saccharin to horse corn, calls it sugar corn, and it satisfies us. We demand of our baker bread containing 35 or 40 per cent of moisture, half-baked and hot, or we eat biscuit instead of eating well baked bread with only 15 or 20 per cent of moisture in it. We take wheat and rice with their rich brown covering of protein and mineral matter and scour off these valuable bone, muscle, and sinew building nutrients which are fed to farm live stock, and we eat the remaining starch which is the heat, energy, and fat building carbohydrate. And so I could go on ad infinitum, but you don't need to hear more. We should select foodstuffs with some knowledge of food values and not with the eye.

We have been living in an age of superlatives and exaggerations. This is seen upon every label of every package of food we buy in one way or another. It is seen in billboards and newspaper advertising. It is heard from the cure-all patent medicine fakir to the crown of thorns and cross of gold statesman. We like it. We demand it. The manufacturer knows we will not listen to a plain, sane, positive degree adjective which could be used in good taste to describe his product. Hence he uses, and with a considerable degree of justification, exaggerated phrases that are declared by law to be false and misleading.

Not only have we ordinary mortals unknowingly helped to make the conditions described, but others whose business it is to understand dietetics and disease have done so by maintaining an attitude of indifference to the problem. I wrote a letter a few weeks ago to each of thirty-five hospitals in Texas inquiring whether they have had a contract with their dairymen as to the manner of the production and handling of the milk which they buy for their sick patients. Not one of these homes for the sick has any such a contract. And some of them to my own knowledge choose to buy cheap, dirty milk rather than the little more expensive clean milk. Then why should a dairyman have a cause to produce clean milk?

In conclusion of this division of my subject, I would therefore ask, Is the manufacturer the only factor in the production of wholesome food?

Did you ever take a calf which you wanted to take the prize at the fat stock show and train it to adopt four mothers and go to eating and growing? It has been done. When it gets old enough, it will need some hay, silage, cotton seed meal and bran. If these feeding stuffs are fed to it in a clean and undecomposed condition, Old Buck will stand a chance to be decorated with a blue ribbon.

On the other hand, if you give him musty hay, rotten meal and bran and a filthy place to live in, in place of a prize winner you will have nothing but a lousy calf.

The same general facts obtain with respect to the man animal. I can take any thin, ugly, knotty, wrinkled faced, despairing woman, and in four months by proper feeding and care brighten up her eye, put the blush of the peach upon her cheek, give elasticity to her step, put hope in her breast, and in short, transform her into "linked sweetness long drawn out"—an efficient woman capable of living an efficient life.

It is a fundamental principle of dietetics that a decayed foodstuff has no food value for any form of animal life. It, however, is a food for vegetable life—a fertilizer only. This fact reveals the love and wisdom of an all wise Providence in Her conservation of forces necessary to the maintenance of life.

It is another well recognized principle of dietetics that a filthy food product, that is, one contaminated with putrefactive bacteria, will rapidly decay under favorable conditions of temperature and moisture either before it is consumed or after it enters the digestive tube; and to the extent of its decomposition it is useless as food for any sort of animal life.

It is furthermore a well known fact that the products of decayed foodstuffs are actual poisons to animal life, the degree of toxicity being proportionate to the degree of the animal organization. Just why the dog does not suffer so much as man for eating a rotten piece of meet is explained upon the principles of *immunity,* which I shall not stop to explain in this connection.

A recognition of these principles is the much talked of pure food law, which declares that it shall be unlawful for a man to sell for food any filthy, decomposed or putrid animal or vegetable substance whether manufactured or not. Such foodstuffs impoverish the animal organism, poison its cells, destroy its health and beauty (which lessens its happiness), and thereby lessens its efficiency.

Is it not just as important to teach these well known simple principles as it is to teach that "all Gaul was divided into the parts," that "thence somebody marched three parasanges," that a Roman praetor performed such and such duties? Should not the State encourage us to spend more time determining what to teach and how much of it to teach rather than asking us to teach a multiplicity of subjects which we know only in a superficial way?

---

In case of intractable "dyspepsia" persistent tenderness in the right iliac region is suggestive of chronic appendicitis as the cause.

